# The experiences of caregivers of children living with HIV and AIDS in Uganda: a qualitative study

**DOI:** 10.1186/s12992-017-0294-9

**Published:** 2017-09-12

**Authors:** Joseph Osafo, Birthe Loa Knizek, James Mugisha, Eugene Kinyanda

**Affiliations:** 1 Department of Psychology, Centre for Suicide and Violence Research , School of Social Sciences, University of Ghana, P. O. Box LG84, Legon, Ghana; 2 Department of Mental Health, Faculty of Medicine and Health Science, Norwegian University of Science and Technology, Trondheim, Norway; 3 Kyambogo University, Kampala, Uganda; 4 Butabika National Psychiatric Referral Hospital, P.o.Box 7017, Off Old Port Bell, Kampala, Uganda; 5 Mental Health Project, Medical Research Council/Uganda Virus Research Institute (MRC/UVRI). Uganda Research Unit on AIDS, Uganda and Senior Wellcome Trust Fellowship, Kampala, Uganda

**Keywords:** Experiences, Caregivers, Children with HIV, Uganda

## Abstract

**Background:**

Home-based care for HIV patients is popular in contexts severely affected by the epidemic and exacts a heavy toll on caregivers. This study aimed at understanding the experiences of caregivers and their survival strategies.

**Methods:**

A total of 18 caregivers (3 males and 15 females) were interviewed using a semi-structured interview guide, and thematic analysis was used to analyse the data.

**Results:**

Analysis suggests that the caregivers are burdened with insecure provisions for food and difficulties in accessing health care. They however survived these strains through managing their relationships, sharing burden with care-recipients, social networks and instrumental spirituality. These findings are discussed under two major themes: 1). *Labour of caregiving* and *2). Survivalism*.

**Conclusions:**

Home-based care presents huge opportunities for community response to the HIV/AIDS epidemic in African settings. It is however burdensome and thus should not be left for families alone to shoulder. There is therefore an urgent need for protecting home-based care for HIV children in Uganda. Implications for improving and strengthening social interventions in home-based care of HIV/AIDS in the Ugandan context are addressed.

## Background

The HIV/AIDS epidemic in sub-Saharan Africa has had both direct and indirect effects on the population. Caregivers of HIV infected persons have been severely affected by the epidemic [1, 2]. The HIV epidemic has for a long time been one of the primary causes of both child and adult mortality in HIV endemic settings within Sub-Saharan Africa including Uganda [3]. This has placed an enormous care-giving burden on the extended family. This burden is particularly heavy on older persons who have a double burden of caring for their sick and dying adult children, as well as orphaned grandchildren [2]. Depending on the intensity of caregiving, such involvement has been observed to negatively affect their domestic economy, health, physical and psychological wellbeing of older caregivers and to lead to short-term weight loss, physical pain, and depression [1, 3–5]. An important part of the discourse is how the epidemic is affecting caregivers (affected) since in resource-poor settings family-centered approaches to HIV/AIDS care are being encouraged [6]. Such calls find relevance in Sub-Saharan Africa, where home-based care for HIV/AIDS patients plays a critically important role for millions of patients [7]. However, family-centered approach of caring for HIV and AIDS persons exacts a heavy burden on the caregivers [8]. Such burdens include physical health deterioration [9], burnout, emotional distress, family breakdown [10–13] and the destruction of household economies [14].

The impact of HIV/AIDS and how it has disrupted family structure and affected the roles of family members including elderly caregivers in Sub-Saharan Africa has been extensively examined [15]. For instance, an ethnographic study in two semi-rural communities in South Africa, which examined the experiences of informal caregivers of people living with HIV, reported that the caregivers, who were predominantly women, were poor, unemployed and unmarried, and combined their care-giving role with their traditional roles as homemaker, household head and breadwinner [9]. The report further indicated that these women experienced physical strains and emotional problems, with risks for being infected with HIV and TB [9]. In this study, there was a preponderance of women and the near absence of men was explained as resulting from the inflexible traditional gendered divisions of labour. The critical need of ensuring the psychological wellbeing of such vulnerable women seems important in informing policies on home-based care [9]. Other studies have reported on the mental health distress of caregivers of HIV and AIDS in the Niger Delta region of Nigeria where caregivers were found to be experiencing stress, anxiety, depression and suicidal tendencies [16]. The findings suggest that there is increasing level of stress in caregiving and requires adequate attention to understand and help reduce this stress. The situation is similar in Kinshasa, DR Congo. A report showed that caregivers’ self-reported health status (predominantly women) was generally poor and they experienced a great burden from caregiving [11]. Other older and young female caregivers in Botswana, have reported feeling overwhelmed with the demands of caregiving [10]. They further reported psychological and emotional difficulties including feeling exhausted, depressed, and often neglected to attend to their own health. In Uganda, elderly caregivers over 50 years and above have reported caregiving burdens including drastic disruptions of living arrangements, prolonged travels and absences from their homes [17].

The burden of caregiving has not changed, although anti-retroviral drugs and treatment have burgeoned. Continuously, the care and support of people living with HIV/AIDS is a major challenge for their caregivers. The Joint United Nations Programme on AIDS (UNAIDS) estimates that 90% of the over 2 million children living with HIV globally reside in sub-Saharan Africa^1^. In Uganda, the country with the fifth highest prevalence rate in the region, 150,000 of the 1.2 million people living with HIV are under 15 years of age. In addition to the physical impact of HIV, epidemiological studies from high-income countries have established that HIV-infected children and adolescents (CA-HIV) experience high rates of psychiatric disorders (PD) with estimates ranging between 27 and 61% [18–22]. African studies on the prevalence of psychiatric disorder (PD) among children and adolescents living with HIV shows that 51.2% of CA-HIV in Kampala Uganda, exhibited above threshold psychological distress scores [23]. The most common diagnoses were depression, anxiety and somatization. In a more recent Kenyan study, Kamau and colleagues reported a rate of 48.8% for at least one PD [24]. PD among children and adolescents with HIV (CA-HIV) not only leads to psychological distress and impaired quality of life both for them and their family, it may also lead to negative clinical and behavioural outcomes for their primary caregivers [8].

In an attempt to understand the new conceptual framework for home-based care in sub-Saharan Africa, studies indicate partnerships between caregivers/patients and more influential groupings as a strategic step [8]. However, the policy documents give no indication on how to mobilize the already over-burdened caregivers and their terminally ill patients. While a lot of research has been undertaken to understand the emotional, social and medical effects of being an HIV career in sub-Saharan Africa including Uganda, the rapidly evolving health policy and HIV care environment in sub-Saharan Africa calls for more up to date studies on how these are impacting home based care. Two recent health policy and health care changes in Uganda and in the rest of sub-Saharan Africa are bound to have had a significant impact on home based care for HIV. First, in 2010 the Ministry of Health of Uganda moved away from a vertical HIV care programme to one that integrated HIV care into the existing health care system. The former was thought to be too expensive and not sustainable in the long term, given the downturn in the global economic environment which had negatively impacted donor funding [25]. Under this vertical HIV care programme, funds mainly from donors were used to support HIV care directly in Uganda. This made HIV care services superior to the rest of the medical services in the country as patients living with HIV (PLWH) had access to: special HIV clinics which were run by designated HIV care physicians and nurses, individualised counselling services, food supplementation, home visitation, and support for children in the form of school fees. Under integrated care, these extra funds were no longer available. PLWH were now exposed to shortcomings of the general medical health system- shortage of health workers, long waiting lines, loss of food supplements, shortage of drugs in general, a deterioration in the care they were receiving [26]. This programmatic change in HIV care has largely been completed in Uganda but we do not know how home-based care for children and adolescents has fared since these changes were implemented, our paper informs this knowledge gap. Second, in 2015, the WHO recommended that all people living with HIV should be offered antiretroviral therapy irrespective of their CD4 counts [27]. As a result, the MOH prioritised giving all HIV infected children and adolescents antiretroviral therapy. Indeed, from the CHAKA quantitative data 95.4% (1277/1339) of the children and adolescents in the study were receiving antiretroviral therapy. How this policy of making antiretroviral therapy universally available to all children and adolescents with HIV impacted home based care is still not known.

This present study examined the experiences of caregivers of children living with HIV/AIDS and how they are coping in the post HIV care integration era and in the era where all children and adolescents with HIV are entitled to antiretroviral therapy in Uganda. It is anticipated that this can further our understanding of the dynamics and contexts of home-based caregiving in contemporary Uganda. This will ultimately guide the development of appropriate and sensitive caregiving and social protection intervention programs in the country.

## Methods

### Study design and setting

This research project is a qualitative interview study, a sub-study of the main study, ‘Mental health among HIV infected CHildren and Adolescents in Kampala and Masaka, Uganda (CHAKA Study). The qualitative sub-study was undertaken at four children and adolescent HIV clinics and one psychiatric hospital. A qualitative study was important since we needed to understand the unique experiences of caregivers or HIV/AIDS orphaned children, how they cope and thrive in their context [28]. Two of the HIV clinics were placed in the urban centre of Kampala (JCRC and Nsambya) and two at semi-urban/rural sites in Masaka (TASO and Kitovu Mobile). Butabika National Psychiatric Referral Hospital in Kampala is a tertiary referral hospital with among others a specialised HIV care unit that also treats child and adolescent with HIV/AIDS. Kampala is the capital of Uganda and has an estimated population of approx. 1, 7 million people, while Masaka district has an estimated population of 300,000 [29]. The main source of income in Masaka district is agriculture and farming. The normative context in Uganda is a strong patriarchy [30] and very religious [31] with dominantly Christianity (85.2%) and Islam (12.1%) [32].

### Participants

The participants for this study were caregivers of children who were born with HIV/AIDS between ages 5–11. They were drawn from the HIV care clinics in the study located at Kampala, Masaka and Butabika. Potential participants were sent to the researcher by the clinic nurse on duty at the triage desk for information about the study. They were female (*n* = 15) and male (*n* = 3). Two were below age 30 and the rest were between age 30 to 5.

### Procedures and sampling

Participants were randomly selected as they came in for their ART (Anti-retroviral therapy) appointments. After they went through clinic procedures, a triage nurse referred them to the qualitative researcher for information about the study. Rrecruitment was done from July to November in 2014. Once they agreed, individual interviews were then conducted with caregivers (5-11 year old child) after taking their consent. Interviews were conducted in a private room and audio recorded. Each participant was reimbursed with transport of 10,000/= (approx. 4USD).

Individual face-to-face interviews using semi-structured interview guides were conducted in Luganda and lasted between 30 min and 1h. Questions on the interview guide included exploring demographics, relationship with the child, history of how the child came to acquire the HIV condition, experiences of providing caregiving, vignettes about children living with HIV/AIDS and mental illness. Probes depended on a participant’s experiences and clarity of their narratives. The researcher who conducted the interviews, is well trained with many years of experience in conducting qualitative interviews. She also translated and transcribed them into English language. All the participants accepted to participate by signing consent forms.

### Ethical considerations

Ethical and scientific clearance for this study was sought and obtained from the Research and Ethical Committee of the Uganda Virus Research Institute, the Uganda National Council of Science and Technology and the Science and Ethical Committee of the London School of Hygiene and Tropical Medicine. Signed consent was obtained from all caregivers of all eligible children with HIV. It was made clear to all participants that refusal to participate in this study would not have any negative impact upon the treatment and care of their children. Due to the anticipated psychological distress following the interview all research staff with direct involvement with study participants received training on how to sensitively deliver interviews and handle situations in which sensitive information is disclosed or emotional distress observed. Participants found to have a psychiatric disorder were referred to mental health clinics nearest to them. In cases of psychiatric emergencies, e.g. highly suicidal individuals or individuals with severe depression, the research staff (who were all mental health workers) provided emergency intervention and referral to psychiatric hospitals.

### Analysis

Thematic analysis was adopted to analyse the data. In the present study, the analysis focused on the responses around the experiences of providing caregiving for these children. Analysis first began by an initial interest in gaining a sense of each transcript [33, 34]. This was achieved by careful reading of the transcripts and noting down initial thoughts about each participant. This first part was to do a within-case analysis in each transcript and noting themes. After, these themes were compared from one case to the other across all the transcripts and then themes which were relevant to the research questions were noted. These themes were discussed by the research group and agreed upon. Further, we established the connections between the themes. We ensured that the working themes adequately represented the evidence contained in the data [33, 34]. After clearly defining and categorizing each working theme the analysis proceeded by discussing it at length during research team meetings in order to maintain the integrity of the responses. Finally, compelling quotes were selected from the transcripts which represented lucid elements of our working themes and relevant to our research question [33, 34].

To address the validity of our findings, all authors had a chance to read all transcripts and develop their own codes. Codes from primary author were shared with all authors to scrutinize and compare with their own initial codes. Codes were discussed with co-authors until consensus was reached about which themes represented the narratives properly. Generally, our analysis and interpretation was jointly conducted by all the authors. Such approach is parallel with the recommendation of Creswell and Miller’s [35], of the importance of cross-validation and group interpretation which facilitates analytic rigour and validity of the findings of qualitative studies [36, 37].

## Results

Important demographics of participants of the study are presented in the table below (Table 1):

**Table 1:** Descriptive demographics of participants

Variable	Frequency
Gender	
Male	3
Female	15
Age	
Below 40	4
Above 40	14
Average age	44.
Education	
No education	2
Primary	9
Secondary	5
Post-secondary	2
Occupation	
Farmer	5
Seller	5
Driver	2
Casual laborer	1
Caregiver	1
Hairdresser	1
Unemployed	3
Relations to child	
Father	3
Mother	5
Grandmother (biologically related)	4
Grandmother (unrelated- e.g., step mums, aunties etc)	6
Family size	
Range	3–11
Average family size	4.7
Age of HIV+ child	
Range	5–11
Mean age	8.8

The table shows that in our sample more women than men are into caregiving. Majority of the caregivers are above age 40. Further, majority also had low education (primary and secondary). They were predominantly educated up to primary level (*n* = 9), senior level (*n* = 5) diploma/certificate (*n* = 2) and a few were not formally educated (*n* = 2). In terms of occupation, they were predominantly working in the informal sector, with majority being farmers (*n* = 5) and sellers (food or petty wares, *n* = 5), and the rest were unemployed (*n* = 3) or keeping some menial jobs. Caregiving was also primarily provided by grandmothers, step mothers and aunties compared to biological parents.

The thematic analysis examined difficulties that are experienced by the caregivers and how they coped under two main themes with sub themes: 1) Labour of caregiving (with two subthemes: *insecure employment and provision for food; and healthcare access dynamics*) and 2) Survivalism (with four main subthemes: *managing sustenance, shared burdensomeness, support networks, and religious resources).*

### Labour of caregiving

This theme addresses all the challenges the caregivers experienced in the provision of care for the patients. Two major difficulties were observed. These included *insecure employment and provision for food*, and *healthcare access dynamics*.

#### Insecure employment and provision for food

A common difficulty faced by caregivers was economic issues. The time spent on caregiving made it difficult to spend more time on income -generating activities resulting in being fired. Lack of food was often the sequel to this as explained by one participant taking care of an HIV/AIDS orphan. His only source of livelihood- a motor cycle he manages for someone and gets paid for that, was taken back from him because other family demands took his attention:

“*I would not want to keep his motor cycle because I was not giving him any money. One day I spent the whole day at the court and he told me that if you feel you are not going to work, never bother getting my motor cycle from here. I explained to him what I was going through and by the time I was done with my brother’s case, I found that the motor cycle owner had given it to another person to use it. So, for some time, I have been without a job and I have not gotten another motor cycle yet. This has affected my family so much. There is a time we failed to have food*” (*Male, 02*).

The crux of this narrative is a loss of job. As explained, this participant was juggling caregiving functions for a child living with HIV/AIDS and also seeking to secure a bail for a brother in police custody. Over time, he lost the use of the motor cycle and thus his source of livelihood. It is obvious in this case how the loss of a caregiver’s job, can affect the lives of many other vulnerable dependents.

In another related scenario, the source of livelihood of a caregiver was destroyed making daily provisions and survival difficult as illustrated in the conversation below:

*Resp: “I am not working. I had kiosks but they were demolished. I haven’t been working since last year*

*Int: Hmm, So how do you survive?*

*Resp: I fared on like that.*

*Int: How do you meet your needs?*

*Resp: I do not have peace of mind but I have to live on but it’s not easy”. (Female, 06)*

In most cosmopolitan centers, the crave for urbanization ignores the poor who survive using menial tools. Kiosks are stalls or cabins (mostly made of wood or light metals) that are scattered in most cities of Africa where most poor and unskilled people display their wares and spend their night. Occasionally, mayors may order such littering of the cities with kiosks to be removed or demolished. This appears to be the fate of this participant- the destruction of her kiosks, a source of livelihood, an experience that can be mentally distressing.

In some situations, the caregiver showed that the caregiving is a partnership but in a form in which the father’s role is perceived different from the mother’s: “*When their father goes to work very far and he doesn’t come back, it becomes so hard for me to manage them since I don’t work”. (Female, 09).* In this partnership of caregiving, the father provides the ‘raw material’ for caring and she (primary caregiver) turns that into the ‘real daily caregiving’. A certain top-down relationship is established and if circumstances impede the flow of provisions from the top to the down, the primary provider becomes distressed as she indicated. As she explained, the potential breakdown of provision from the top to the down can be salvaged if the primary provider is gainfully employed.

Some of the participants indicated that lack of food was a major challenge. Though three-square meals a day was impossible, some aimed at a meal, at least a day: *“If I fail to get lunch, I struggle to get supper at least” (Female, 19).* Others indicated that food was scarce and this led to improvising to solve the problem: “*Famine is common and leads you to start looking for food here and there. You try to improvise but often they don’t get satisfied. You think of something different the next day”* (*Female, 07*). The inadequate improvised methods lead to further improvisation, something that can reflect resilience but may also produce helplessness over a long haul.

In some instances, lack of daily provisions generated mental distress. A participant explained how in the past 30 days she has always failed in getting food and became mentally disturbed about this:

“Resp: *Yes I failed always and there was a particular time that I was so confused*

*Int: Tell me about it.*

*Resp: The children were very hungry, yet I did not have a single shilling. I was hungry too, because as you now know I had to swallow my ARVs (Anti-retroviral drugs). I even cried because I did not have any plans in mind. I was confused about what I would do next. So, I sat in my house. As I moved about…fortunately someone gave me 1000/= I used it to buy posho (maize mill) but eating it was also a hassle because I did not have any soup to accompany it”. (Female, 18)*

This is a typical case of double agony: infected-infected scenario. She is a patient and has to deal with her own hunger and medical therapy simultaneously with that of the children. Her mental state at that moment could be gleaned from the indications of *crying*, not *having any plans in mind*, *confused* and *sitting in the house*. When she *moved about,* suggesting she left the house perhaps looking for some help and then someone was benevolent to her but the nutritious quality of the meal was also a challenge (*posho* has a source of carbohydrate but no protein). Thus, there was an overall sense of desperation to get food and nutritious food in her state of poor health. One wonders the extent to which such hassles for food by a vulnerable caregiver can influence her ability for providing caregiving to children who may be more vulnerable than herself.

#### Healthcare access dynamics

This sub-theme examines the challenges related to access to healthcare facilities and some of the dynamics that related to such access: “*At first we went to a faraway hospital. We later gave up because it was so hard to raise transport. Fortunately, we were changed to this nearby hospital”. (Female,13).*

What is explicitly clear in this narrative is that inability to access healthcare in a remote location discouraged regular visits and perhaps, consequently destroyed adherence. The difficulty of access is dynamic as relates to raising transport fares. Access to a nearby health facility is viewed as act of fortune as explained.

Access to the healthcare facility is further hampered by lack of money occurring in tandem with poor physical health as illustrated in the following conversation:

*Int: Have you ever failed to bring the child to hospital in the past 6 months?*

*Resp: Yes, I failed and the doctors scolded me. I explained to them that I could not walk the distance from home to here.*

*Int: Was it money or energy that was lacking?*

*Resp: It was money. The next time I did not have energy; so I came on another day after the appointed date (Female, 15)*

For half a year lack of funds and poor physical health hampered her from visiting the hospital. Noteworthy is the alleged response of the doctors to her absenteeism- she was scolded. The dynamics of access is thus not simply availability of health care and lack of funds, but rather certain peculiar characteristics of the participant such as poor health. Further, the indication that she was met with a poor health worker attitude when she finally reported showed other health-facility and worker-related issues which might conspire with other factors to further increase non-compliance behaviours in caregivers.

### Survivalism

This theme addresses the livelihood strategies by participants in organizing scarce resources as means of reducing the strains of caregiving. The struggles to cope with the demands of caregiving was undergirded by a huge sense of desperation which warranted survival. This is illustrated in the voices of some of the caregivers: “*I struggle as a mother, it cannot happen for us to go without food. Whatever I get, is what we eat” (Female, 12).* Others corroborated this: *I would rather go through thick and thin to make sure we come on every appointment date because health is the most important thing. We want the child to be alive (Female, 10*) and “*If I failed to get lunch, I struggle to provide supper at least” (Female 06).* But for others, their struggle to survive failed*: I give them porridge. I struggle a lot; only that I fail sometimes (Female,17).*

The resources for survival ranged from tangible supports such as money and social support and non-tangible ones which are psychological and spiritual. These are discussed under specific sub-themes such as *managing sustenance*, *shared burdensomeness*, *support networks* and *instrumental spirituality.*

#### Managing sustenance

This sub-theme addresses the skills these caregivers deployed in an attempt to make ends meet. Most of them reported having to fall on managing their relationships at various levels in order to bring in or keep scarce material resources within their reach. Others did manage the scarce resources by liquidating surplus from farm produce. In terms of managing relationships, some kept good rapport with creditors, neighbors, their dependents/patients, school authorities and others. For example:

*The good thing is that I have created rapport with many people in my area having retail shops. I am able to get home requirements from them on credit and pay later. I do the same at the market near where I work from and that is how I have managed to sustain my family. But the best I did was to buy around three kilograms of sugar so they are able to prepare porridge and have that. If I get cassava or sweet potatoes, they eat that with porridge. I tell them not to mind that, if God helps us and I am able to work again, they will be able to feed well like they used to*. (*Male,* 01)

This caregiver manages two main relationships. The first level is to keep a credit worthy-posture with his creditors at both proximal (where he lives) and distal (market he attends) levels. He takes a central stage in this when he said ‘*I have created rapport with many people and that is how I managed to sustain my family’.* This reflects agentic posturing where he shoulders all the responsibility in improvising for their survival. The second level of relationship is domestic, with his dependents, the children he cares for. He manages such relationship through bulk purchase of daily provisions and facilitating hope in the children. This hope in life, and living is fused with religious themes of divine providence in expectation of thriving and returning to a better state of livelihood as it was in the past.

In other cases, the caregiver managed her relationship with creditors to be able to send the child for medical attention: “*It would have happened but whenever I don’t have money, I tell the person who normally transports me to bring me on credit. I then pay later when I get the money*” (*Female, 06*). In this other case, the caregiver has established a rapport with the headmistress of the school of the children so that in the event of non-payment of fees on time, she can be pardoned:

*“…There’s also an organization that the headmistress had got for us that paid his school fees, but they ran out of funds. So, it is his father currently. At times when I call him and he says he doesn’t have money, I sell some of the surplus from the crops we grow, if it is a good season. I take that money to school and explain to the headmistress while asking her to be patient with us until we can top up” (Female, 11).*

What is noteworthy is that she liquidates surplus from the farm produce to offset school bills. The liquidation and managing the relationship with the headmistress in the school appear to be desperate measures, consequential of failure to raise monetary support from the NGO and the father of the kids.

The dynamics of managing insufficient resources sometimes involved gender role tensions. In some situations, the father left the nitty-gritty of caring to the mother in the face of abject pennilessness. In such situations, the woman appeared to have accepted a challenge to search for means of survival as one of them explained:

*I have never failed, when I did not have any money. Their father does not want to hear anything about hospital, yet, he is aware of it. I am to look for the money and bring the child to hospital. I later found out that when you miss coming, the health workers are not happy about it. I do what I can; even if it means to borrow money and bring him*. (*Female, 17).*

This caregiver reported that she has never failed to raise money to transport the child for medical attention. She takes a central stage when the father reneges on his responsibility and her extensive use of the pronoun ‘I’ may emphasize the burden but also the fortitude to thrive in the midst of scarce resources as though she is a single parent. She introduces the poor health-worker attitudes towards her perhaps as a means of juxtaposing her desperation but also fortitude with the ignorance of the health workers in terms of the struggles of the caregiver. This caregiver appears ready to surmount all hurdles in adherence to treatment and perhaps health workers must appreciate such struggles.

In another gender-related case, a father abandons his HIV positive child on the grandparents to provide care. Every attempt from the caregiver (grandmother) to get the father to demonstrate responsibility towards the child has failed; under the pretext of financial crisis:

*We, the grandparents pay the child’s school fees…we take care of him ourselves because the father is not showing any concern for him. There are times we ask him to come over when the child is sick, all he does is promise to come the following day and he never shows up. Claiming that he does not have any money yet (Female, 04)*

This caregiver further illustrates the struggles with food and hospital visitation when she indicated that “*we have food although in small amounts sometimes”* and “*I always struggled as much as possible to ensure that we come (to the hospital)”*. There is outward evidence of thriving but beneath is a sense of desperation, and like other families, they also managed by selling produce from the coffee plantation: “*We pick coffee and dry it. The coffee is then sold to raise money for school fees…” (Female, 04).*

#### Shared burdensomeness

Another way some participants coped with the demands of caregiving was through sharing the burden with the patients. As explained by a male caregiver, the HIV+ children participate in household chores and decision making in terms of menu for the day:

*We help each other; the spring well is very close. What I do, I tell them to fill the water containers and when I come by, I carry the water to the house. The spring well is around twenty meters from my house. I also ask them what they would want to eat, because I do not normally buy a lot of things to keep around. So, I ask the older boy to ask his sister what they would want to eat… So the choice they make is what I go with*… (*Male, 03*).

In some cases, the children participate in cooking and attending to customers patronizing the canteen of a caregiver. The caregiver views such engagement of the children as a normal activity. The question, however, is whether such demands may not put extra burden on their already debilitating health condition:

*We do help one another, when I cook the other washes the dishes. I even ask them to help me to attend to customers in the canteen. I tell him to wash the uniforms. They help just like all the other children. (Female, 08)*

The physical condition of the HIV positive child definitely may not be compared with the normal healthy child, but this is how the caregiver (who is also infected) circumvented the burden of caregiving- sharing the household chores with the infected child and perhaps providing the child with a feeling of being important. A caregiver in one instance felt unenthused to present the child for medical attention and asked the 8 years old child to go in the company of the teenage sister:

*At the place where we get drugs, we used to pay 500/=, we now have to pay 1000/=. Sometimes I can give him the money and ask him to go for the medicine and I don’t go. Sometimes I do not feel energetic enough to move that journey.*

*Int: Where do you get the drugs from?*

*Resp: Nkoni*

*Int: So he goes that far. How does he go?*

*Resp: He goes with the other girl I have told you about. (Female, 07)*

Breaking the household chores into chunks and assigning each child to handle a piece of it was another way some caregivers sought to manage the burden of caregiving: *“From the garden, we all share tasks. For example, one takes the goats to graze, another washes the dish while another peels food with me. That’s how we do it” (Female, 11).* However, in some cases some of the children who are being cared for with caregivers who are positive also provided support to ease their burden as narrated below:

*The children usually tell the others not to bother me saying that my CD4 cells will fall. Even to this child, who is also on ART. They all know that now…At times they remind me to take my medication. They even do not want to disturb me saying; ‘leave grandmother alone, you will lower her CD4 cells (*Cluster of Differentiation 4)*. They know it also. (Female, 15)*

As indicated, these children appear literate about the immunology of HIV/AIDS and provide emotional support for their caregiver and one other child. The support includes prompting the caregiver towards adherence and allowing her serenity of mind. These supports are geared towards protecting the immunity of the caregiver and minimizing her struggles. This is consistent with another study among African-American children who have been found to be social actors of care [37].

#### Support networks

Others have coped by constant provision of support from sponsors. The sponsorship is all-encompassing including the provision of food, school fees, and transportation to present the child to the health facility for attention:

“*I have never failed because that child has got sponsors who give him support and they are the ones that are paying his school fees. They are also the ones that give me money that enable me to bring him to the clinic. They are also the ones who brought him to this clinic”. (Female, 16)*

As indicated above the support the caregiver receives is a whole package; including school fees, payment for drugs, transportation to the health center and similar to others who received support from relatives such as uncles for the payment of fees “*There’s an uncle of hers who pays her school fees” (Female, 12)*.

The above is contrary to the experiences of others, who received partial sponsorship and have to top up in order to survive. Some of such partial sponsorships came from older children of the caregivers as illustrated here: *My daughter often sends me some money. I also sell the surplus from my garden to raise more money to get money that we can use at home. (Female, 07).*

Others receive meagre support from other family members such as grandfathers and were barely surviving on this:

*Int: Who provides food in the home?*

*Resp: Their grandfather.*

*Int: Who pays school fees for this child we are talking about?*

*Resp: His grandfather, but his income is very low. He pays it, but in a lot of agony. He *

*doesn’t have any stable source of income. (female, 40)*

The burden of caregiving is laid on the grandfather who might be retired and thus unemployed. As implied, such demands stretch him, this can emotionally, and physical wear him out.

#### Instrumental spirituality

Religious resources also provided means of coping with the demands of caregiver burden. These resources are materialized in the way religious rituals such as prayer are viewed as instruments for receiving the provisions for livelihood as explained by a participant: *I asked God to give me a job where I can get school fees for my children.* Later when he was asked whether food has been scarce for the family his response reflects a religious expression of confidence that God provides for their needs:

*Int: In the past thirty days; has it ever happened that there was no food in the house?*

*Resp: No, we have food all the time. God has been providing for us. (Male, 02)*

The participant relies on prayer to solve an employment and educational need. His faith is still instrumental in the way he expressed faith that the care they receive from the divine is practically expressed in their daily provisions. This sense of hope and faith in God in aiding their thriving is an undercurrent and a reflection of the extent to which the utility of religion is widespread in his survival tactics.

Prayer was used by others in procuring food for daily living. The nature of the answered prayer is however non-super naturalized. It is rather re-ordering of the participant’s social environment in a manner that fostered sustenance, thus, someone provided help:

*“There was a time we lacked food, but when we prayed, our prayers were answered.*

*Somebody randomly offered me sweet potatoes. Shortly afterwards, I noticed a child crossing the road heading towards me with beans. You know, I stay in a village setting trading center; when those people harvest, they share with the neighbors. Personally, I don’t have a garden. The Lord came to my rescue, but I really didn’t have food”. (Female, 10).*

The events following the prayer are viewed as favorable response to the prayer. From the narrative, the neighbours who offered the potatoes and beans all appeared to have been on a divine mission. Such thought is clearer in the face of the practice of reciprocal benevolence present in the village: people harvest and share. The implication as the participant explains is that, if you have a garden then you can be a part of this practice, but she does not have one. In this scenario, what will influence someone to give to another is beyond any social exchange ethic. The participant believes prayer, a supernatural instrumentation, influenced the benevolent acts that was extended to her from some community members. In the African context, the mundane and the divine are inseparable and this is what appears to be indicated here [38].

## Discussion

The purpose of this study was to investigate the experiences of caregivers of HIV+ children and how they cope with the demands of caregiving. To begin with, the demographics of the caregivers in the present study are consistent with the reports of other studies about particular demographics of caregivers of HIV children. First, majority of these caregivers are within the age period where they can leverage their energies on caring demands. However, if they do not get support, all their energies can be spent on the demands of caring leading to burnout and other psychological problems [15]. In the main study, 19.6% of the caregivers had significant psychological distress scores on the SRQ-20 (Self-Reporting Questionnaire 20-item). Second, the low level of education among the caregivers is disturbing since it could hamper access to health information and perhaps adherence to anti-retroviral therapy. In one study for example, although self-efficacy moderated the relationship between literacy level and adherence to treatment regimen, a multivariate analysis showed that low literate persons were 3.3 times more likely to be non-adherent to their antiretroviral regimen [39]. Further, most of the caregivers were engaged in marginal jobs which are lowest paid and insecure [40]. Such households may be economically vulnerable and the children may live with heightened risks of malnourishment, consistent with other studies in Nigeria [41]. Additionally, caregiving was essentially provided by more members of the extended family folks including grandmothers and aunties. Some studies have also reported that grandmothers (and for that matter extended family relations) are active care providers for children living with HIV [42]. There are also gender dimensions in the demography of these caregivers, which cannot be overlooked. Majority of the caregivers were women and that might define caring primarily as female endeavor [43]. This is consistent with the literature on caring and specifically caring for HIV and AIDS patients where women are cited as the major resource in informal home-based care [44]. Most studies explain that men and boys may be underrepresented in the caring repertoire of HIV and AIDS patients due to the social norms and role expectations that view caregiving as women’s responsibility. Consequently, home-based care by men may be unacceptable in most African communities [9, 45]. However, such representation of caregiving has serious implications for the economic empowerment of women. In the report on HIV care in Zambia, Augestina Chella, the VSO Programme Manager, indicated that the burden of HIV & AIDS care has dehumanized women, and virtually feminised poverty and turned women into work horses in the name of volunteering and caring for the community [46]. These demographics generally point towards a potentially fragile family resources in terms of gender, low education, insecure jobs and perhaps the depletion of psychosocial energy through the demands of caregiving.

There is no doubt that caregivers in this study are playing a critical role in communities’ readiness to respond to the HIV epidemic [47]. The first important indication is that home-based care for children living with HIV is pervasive and leaves the onerous responsibility of caregiving on the mothers or family relatives. Yet, the families’ domestic economies are unable to support the caregiving demands for such children. For instance, the indications that these caregivers are struggling with finances, food, school fees, transportation problems to healthcare facilities are consistent with other studies in Botswana [47], Namibia [48], South Africa [42, 49], Zimbabwe [50] and Ethiopia [51]. The sum total of all the strains of these caregivers converge on one fact: family caregivers who are major stakeholders in non-institutionalized services for HIV patients in Sub-Saharan Africa are burdened from the duties of caregiving [52]. This burden on caregivers might influence poor caregiver health [53]. HIV/AIDs is a chronic condition and these caregivers might be involved in long-term informal caregiving with its attendant demands for daily provisions such as food and healthcare as distractions. These competing responsibilities may lead to delay in seeking self-care; placing low priority on their own health [54, 55]. If these caregivers break down, this can represent a double burden, a real threat to family resilience.

The findings further showed various dimensions of coping with the struggles of providing caregiving for HIV/AIDS orphaned children in Uganda. Firstly, HIV caregivers in this sample, improvise in many ways to survive the strains of caregiving. These included managing their sustenance, shared burdensomeness, social networks and instrumental spirituality. In a study conducted on older caregivers living in rural Kenya, participants demonstrated ability to mobilize new resources of labour for food production and formed new social networks in order to foster other forms of food entitlement [56]. Further, Wangui reported that caregivers also received credit from the social networks when people they knew donated food directly or indirectly received money to buy food. Such reports are consistent with our findings where we observed caregivers managing their sustenance and social networks in ways that strategically ensured the constant supply of basic provisions such as food. As extensively reported by Ntozi and Nakayiwa [57], home-based care of HIV patients by relatives in Uganda is not a recent phenomenon. The struggle to cope does reflect the thriving of extended family safety net mechanism in dealing with the burden of HIV/AIDS on the family [58]. Without romanticizing their strengths in the face of the struggles for caregiving, it can be arguably defended in this study that the caregiving of HIV/AIDS orphans to some extent reflects family resilience in Uganda [59]. The burden however, is ever increasing, dramatically changing family structure and threatening household resource [58, 59].

Secondly, generally, caregiving is assumed to be uni-directional, meaning the care-recipient (in this case children) are passive and support is flowing from the caregiver in a linear fashion to the receiver [60]. However, as Fergus and colleagues [61] argued, this may be erroneous since the care-recipient is an active agent who supports the caregiver’s efforts. Such reciprocation has received limited attention in the literature [62]. The sharing of burden with care-recipients as observed in this study may represent forms of reciprocation and patient-provided support in ways of easing the impact of caregiving burden. In this regard and many other reasons, we can conceptualize the support children provided for these caregivers as crude means of alleviating their stressors in the context of dearth of institutional interventions. Such posture from the children can assuage the attention of the caregivers to the positive dimensions of caregiving as pointed by Walker and colleagues [62]. The present study may thus be one of many attempts to show and document such reciprocation in the caregiver-care receiver dyad in Uganda.

Spirituality represents another dimension of coping with the demands of caregiving in this study. Spiritual support focuses on a person’s internalized resource that flows from the perception of an intimate relationship with a higher power [63]. Spirituality has repeatedly been found to be inversely associated with mental health dimensions including low self-esteem, depression, loss of meaning in life, hopelessness, etcetera [64]. There is evidence of high rates of religiousness and religious coping among informal caregivers of all kinds of care-recipients and some associations with better mental health outcomes [65]. The use of prayer in this study as a survival strategy for some caregivers was instrumental in accessing daily provisions such as food. Such material utility of prayer sharply diverges from another study, for instance, in the USA among HIV caregivers where prayer was generally deployed to gain psychological composure in caregiving including managing positive emotions such as gratitude, trust, faith, gain focus, calmness, guidance and moral direction [66]. In Africa, religion and life (both private and public) are so deeply fused and the material utility of religion in search for solutions to life’s crises are everyday experience, especially with the upsurge in Pentecostal and neo-Pentecostal churches [67, 68]. As rightly observed by Asamoah-Gyadu, “…the quick African resort to the sphere of religion in the search for solutions to life's difficulties” (p.93). The use of prayer by the caregivers is consistent with such assertion and parallels other reports of caregivers of HIV persons in the USA who used prayer to cope with the demands of caring [68]. This has implications for the role of religion and the church in the management of home-based care services for families affected with HIV.

A closer reflection on these struggles and survival strategies of these caregivers, point towards a general experience and sense of desperation for surviving the impact of HIV/AIDS on families in Uganda. Compared with other studies elsewhere, such as Burkina Faso, caregivers’ challenges were more psycho-emotional, dealing with stigma, routine difficulties of taking the pills, difficulties relating to disclosure of HIV status to children and family folks, and eventual sense of isolation from social support that may be needed [69, 70]. What we found in this study is difficulties related to basic human needs. Although home-based care comes in handy in the face of scarce social interventions, it results in the depletion of household resources, making daily provisions difficult to come by. To surmount these struggles, caregivers in this study needed to negotiate their relationships in ways that facilitate thriving. Although such survival strategies may be salutogenic and be commendable within the discourse of human agency and resilience, the consequence of virtually institutionalizing a culture of poverty among these groups of caregivers in Uganda looms large, and the urgent need for social interventions cannot be overemphasised.

## Conclusions

The study has elucidated the burden of caregiving and survival strategies of caregivers of children living with HIV in Uganda. Their struggles have been found to be centered on material scarcity such as food and funds for daily provisions. The survival strategies concretely reflect desperate efforts towards livelihood and illustrate the culture of poverty among HIV caregivers as reported by some studies [11, 71]. The findings have two important implications for interventions.

First, direct social intervention programs from government (in terms of daily provisions such as food) to resource-limited caregiving families of HIV patients is urgently required if informal home-based care for HIV should be a viable alternative or addition to facility-based services in Uganda. The current facility-based care model in the country that is essentially the provision of drug refills, counselling and peer activities, should be expanded to include direct supply of necessities such as food to vulnerable families. The Social Protection Program under the auspices of the Ministry of Gender, Labour and Social Protection, could be extended to include Home-based care providers of HIV and AIDS orphaned children and adolescents. Currently, there is no Social protection policy for Home-based caregivers in Uganda and this study draws into sharp focus the urgent need to consider this group as needful for such services. The piloting of the Senior Citizens Grants (SCG) in six districts can be targeted to include senior citizens who are engaged in Home-based care for children living with HIV and AIDS. The key targets of the HIV report in 2013 of Uganda including eliminating gender inequalities, eliminating stigma and discrimination and strengthening HIV integration were significantly reached. However, clear targets of practical provision of social support and protection such as providing basic needs for survival such as food for PLHIV and HIV affected families is far from the targets. Indeed, there is a provision in the 2011–2015 National Strategic Program Plan of Interventions (NSPPI) which focuses on reaching about 51% of all Ugandan children considered to be critically in need and vulnerable. One key objective of the NSPPI is to empower the capacity of caregivers as well as protection and provision of care for orphans and other vulnerable children [28]. The findings in this study urgently bring this objective into clear focus.

Second, women who primarily provide care for HIV patients in Africa need social protection and intervention programs to support them. Relatedly, government should put in place intervention packages with attractive incentives for neighbours and family relatives to take up paid home-based care [40]. Thus, home-based care should be strengthened [8]. As recommended elsewhere, the provisions of community-based paid care should be encouraged, similar to the Integrated Community-based Home Care model in South Africa [71]. The study contributes towards the urgent need for protecting home-based care for HIV children in Uganda and in this regard, the advice from D’Cruz [48] is instructive:

“The world over, as families shoulder the responsibility of providing care to their seropositive loved ones, McGrath et al. [72] warn that in the absence of adequate support, families will be unable to cope. According to them, the loss of equilibrium could precipitate a sense of disintegration, which in turn could call into question the future of the family as an institution” (p. 200).

### Limitation

While this study was undertaken within the new policy environment where HIV care has moved from vertical care programmes to integrated programmes, it did not set out specifically to look at the impart of this policy shift on home based care. Therefore, there is the need for specific studies to investigate the impact of this programmatic shift on home based care for children and adolescents with HIV in order to inform future programme development. Further, most of the caregivers were women. Efforts should have been made to get more males who are engaged in caregiving to share their experiences. Although caregiving appears gendered in most African settings, the presence of men in informal and formal caregiving cannot be denied.

## Abbreviations

ART: Anti-retroviral therapy
ARVs: Anti-retroviral drugs
CD4: Cluster of differentiation 4
CHAKA: CHildren and Adolescents in Kampala and Masaka
JCRC: Joint Clinical Research Centre
MRC/UVRI: Medical Research Council/Uganda Virus Research Institute
SRQ-20: Self-Reporting Questionnaire 20-item
TASO: The AIDS Support Organization
